# Distribution, karyomorphology, and morphology of *Aspidistra subrotata* (Asparagaceae) at different ploidy levels in limestone areas of Asia

**DOI:** 10.3897/CompCytogen.v11i1.9803

**Published:** 2017-01-09

**Authors:** Jian-she Chen, Qi Gao, Hao Zhou, Yu-song Huang, Mikinori Ogisu, Ming Cao

**Affiliations:** 1 Guangxi Key Laboratory of Plant Conservation and Restoration Ecology in Karst Terrain, Guangxi Institute of Botany, Guangxi Zhuang Autonomous Region and Chinese Academy of Sciences, Guilin 541006, China; 2 1-30-43 Kamitakaido, Suginami-Ku, Tokyo 1680074, Japan

**Keywords:** Aspidistra, chromosome number, karst plants, karyotype asymmetry, polyploid complex

## Abstract

*Aspidistra subrotata* Y. Wan & C.C. Huang, 1987 is considered for the first time as a widespread polyploidy complex in the genus *Aspidistra* Ker Gawler, 1823 from limestone areas of Asia. The chromosome number of the tetraploid is 2n = 76 and the karyotype is formulated as 2n = 44 m + 8 sm + 24 st, while the chromosome number of the diploid is 2n = 38 and the karyotype formula 2n = 22 m + 4 sm + 12 st. In our studies, diploids occupy broader geographical and environmental niche spaces than tetraploids. Although the leaf-shape of *Aspidistra subrotata* varies quantitatively between and within diploid and/or tetraploid population(s), no obvious discontinuity in the width of leaf has been observed. The tetraploid plants may be distinguished from the diploid plants by their rigid petioles as well as thick deep green lamina. *Aspidistra subrotata* is therefore an interesting material to explore the formation and the evolutionary dynamics of a natural polyploid complex from limestone areas of the tropical regions.

## Introduction

*Aspidistra* Ker Gawler, 1823 is a large genus including more than 140 species from Asia, belonging to the Asparagacae (Li 2004, Tillich 2014, Zhou et al. 2016, APG IV 2016). Its species diversity center is distributed in southwest China and northern Vietnam. Most of the *Aspidistra* species are diploid, with chromosome numbers of 2n = 36 or 2n = 38, except only two tetraploid species, *Aspidistra xilinensis* Y. Wan & X.H. Lu,1987 (2n = 72) and *Aspidistra cruciformis* Y. Wan & X.H. Lu, 1987 (2n = 72), both from China, as well as one hexaploid species, *Aspidistra sutepensis* K. Larsen, 1961 (2n = 114) from Thailand (Qiao et al. 2008, Meng and Gao 2014, Gao et al. 2015). All of these polyploids are stenochoric species with a chromosome base number of x = 19.

*Aspidistra subrotata* Y. Wan & C.C. Huang, 1987 was originally found in Guangxi Botanical Garden of Medicinal Plants, Nanning City, Guangxi Province, China. After that, Huang et al. (1997) reported on the chromosome number of *Aspidistra subrotata* from Nanning 2n = 38, with a karyotype formula as 2n = 22 m + 2 sm + 14 st (2 sat), while Wang et al. (2000) reported the same chromosome number in plants from Guilin City, Guangxi Province, China, but with different formulae, 2n = 22 m + 6 sm(2 sat) + 10 st. Both of the above plants were cultivated in the Botanical Garden and their wild localities remain unknown. Aspidistra subrotata subsp. crassinervis Tillich, 2005 was found from Vietnam, possibly in Tam Dao, Thai Nguyen Province, which was distinguished by Aspidistra subrotata subsp. subrotata in having its lamina ovate–lanceolate, secondary veins sharply protruding on upper surface of lamina, perigone lobes red–purple, ant the stigma white or with few small red dots on its upper surface. It is noteworthy that Tillich (2005) also pointed out that Aspidistra subrotata subsp. subrotata could be collected from the same locality. When studying the diversity in leaf shape of Thai material of *Aspidistra subrotata*, Phonsena and De Wilde (2010) recognized that plants with narrow or broad leaves with a smooth surface or with broad leaves and raised nerves were not found inter-connected with their rhizomes, although they usually grew in the same population. Therefore, three varieties were confirmed: Aspidistra subrotata var. subrotata with leaves 4–7 cm wide and nerves not raised, Aspidistra subrotata var. angustifolia Phonsena, 2010 with lanceolate leaves (1–) 2–2.5 cm wide and nerves not raised, and Aspidistra subrotata var. crassinervis (Tillich, 2005) Phonsena, 2010 with leaves 4–7 cm wide and raised nerves. During our systematic study on *Aspidistra*, we have conducted field work in China and Vietnam, and studied the karyomorphology and external morphology of *Aspidistra subrotata*. The tetraploid populations of *Aspidistra subrotata* were reported here for the first time, as well as recording widespread polyploidy complex in the genus *Aspidistra* from the limestone areas of Asia. This study is mainly aimed to add our knowledge about cytology of this genus.

## Material and methods

The plants were collected from field work in Guangxi Province, China and Hanoi, Vietnam (Table 1) and subsequently cultivated in the experimental garden of Guangxi Institute of Botany, Guilin. Only one sample from Guangxi Botanical Garden of Medicinal Plants may be the clone plants of typical *Aspidistra subrotata*. For chromosome observation, actively growing root tips were pretreated in 0.1% colchicine for 3 h at room temperature and then fixed in Carnoy I (ethanol : glacial acetic acid = 3 : 1). They were macerated in 1 : 1 mixture of 1 M HCL and 45% acetic acid at 60 °C for 4 min, and stained and squashed in 1% aceto-orcein. The karyotype formula was based on measurements of metaphase chromosomes taken from photographs. The symbols used to describe the karyotypes followed by Levan et al. (1964). From the mean values of one to five individual karyotypes, the average chromosome length as well as the karyotype intrachromosomal asymmetry index (A_1_) and interchromosomal asymmetry index (A_2_) (Romero Zacro 1986) were calculated.

**Table 1.** Material examined of *Aspidistra subrotata*.

Sample	Voucher	Location	Latitude Longitude	Altitude	Figure
JL	*Huang Y.S. QG375*	China: Jinlong Town, Longzhou County, Chongzuo City, Guangxi Province	22°26.04'N 107°01.65'E	ca. 300m	
5M	*Huang Y.S. QG378*	China: 5th boundary marker, Nonggang National Nature Reserve, Longzhou County, Chongzuo City, Guangxi Province	22°27.82'N 106°58.03'E	ca. 300m	
NG	*Wu W.H. QG526*	China: Nonggang National Nature Reserve, Longzhou County, Chongzuo City, Guangxi Province†	22°28'N 106°58'E	~	5a
NN	*Gao Q. QG735*	China: cultivated in Guangxi Botanical Garden of Medicinal Plants, Nanning City, Guangxi Province		~	5b
SK	*Anonymous QG766*	China: Shuikou Town, Longzhou County, Chongzuo City, Guangxi Province†	22°28'N 106°35'E	~	
4M1	*Gao Q. QG807*	China: 4th boundary marker, Nonggang National Nature Reserve, Longzhou County, Chongzuo City, Guangxi Province	22°27.61'N 106°57.95'E	ca. 400m	6a
4M2	*Gao Q. QG809*	China: 4th boundary marker, Nonggang National Nature Reserve, Longzhou County, Chongzuo City, Guangxi Province	22°27.58'N 106°57.93'E	ca. 370m	6c
4M3	*Gao Q. QG810*	China: 4th boundary marker, Nonggang National Nature Reserve, Longzhou County, Chongzuo City, Guangxi Province	22°27.57'N 106°57.93'E	ca. 370m	6e
4M4	*Gao Q. QG811*	China: 4th boundary marker, Nonggang National Nature Reserve, Longzhou County, Chongzuo City, Guangxi Province	22°27.53'N 106°57.93'E	ca. 380m	5c, 6h
PM	*Liu Y. QG281*	China: Mt. Poman, Napo Town, Baise City, Guangxi Province†	22°57'N 160°00'E	~	
DQ1	*Liao Y.B. QG662*	China: Mt. Daqing, Pingxiang County-level City, Guangxi Province	22°18.27'N 106°41.93'E	ca. 950m	5f, 7f
DQ2	*Gao Q. QG823*	China: Mt. Daqing, Pingxiang County-level City, Guangxi Province	22°18.06'N 106°42.15'E	ca. 900m	7a
BV	*Ogisu M. QG365*	Vietnam: Mt. Bavi, Hanoi†	21°05'N 105°22'E	ca. 540m	5d, e

† Lack of exact GPS

## Results

### Distribution and karyomorphology

Karyomorphological features were observed in 13 samples of *Aspidistra subrotata* (Table 1). Of these, eight samples of five populations from Longzhou County and one sample from Nanning were tetraploid, while the other four diploid samples were from Mt. Daqing, Pingxiang County-level City, Mt. Poman, Baise City and Mt. Bavi, Hanoi.

**Map 1.**
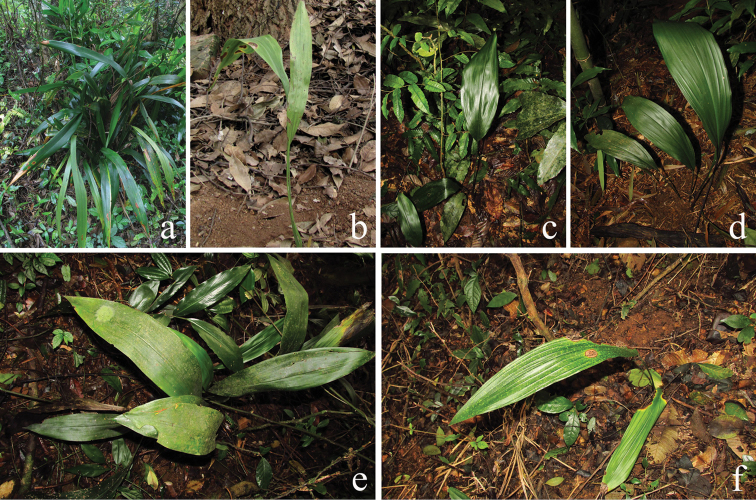
Distribution of *Aspidistra subrotata*. Black circle represents the tetraploid population; gray circle represents the diploid population; empty triangle represents typical Aspidistra subrotata var. crassinervis; empty square represents typical Aspidistra subrotata var. angustifolia.

**Figure 1.**
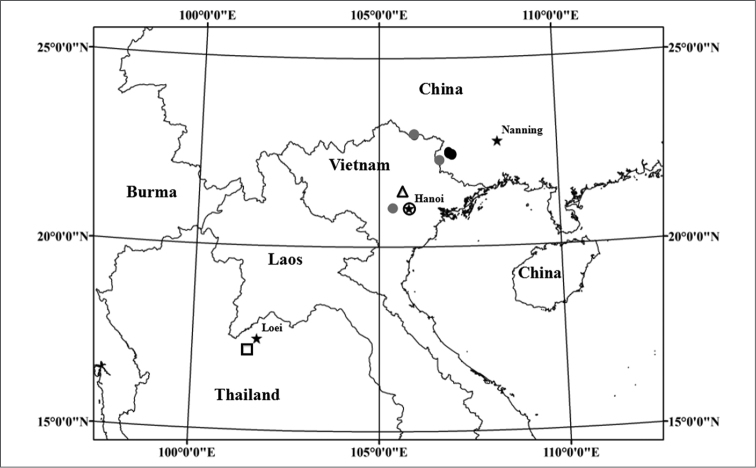
Somatic Chromosome at mitotic metaphase in *Aspidistra subrotata* of 2n = 76. **a** Jilong population **b** 5th boundary marker population **c** Nonggang National Nature Reserve population, and the arrow shows the secondary constriction of the chromosome **d** 4th boundary marker population. Bar = 10 μm.

Eight samples of five tetrapoloid populations of *Aspidistra subrotata* were all from Longzhou and were located not far away from each other (Map 1). Another single sample from Nanning was cultivated in Guangxi Medicine Botany Garden, which may be the clone plants of typical *Aspidistra subrotata*. All of them have a chromosome number of 2n = 76, with the karyotype formulated as 2n = 44 m + 8 sm + 24 st (Figure 1, 3). The karyotypes of the tetraploid samples are similar to one another. The first and largest pair was metacentric. The pair II was larger [larger than what?]and submetacentric. Chromosomes from III to VIII pair were larger [again, larger than what? Normally, these chromosome pairs would be expected to be progressively smaller] and submetacentric. The other chromosomes were smaller, of which pair IX was submetacentric and the others were metacentric (Figure 3). It was noted that the secondary constriction occurred in chromosome 12 of the sample NG (Figure 1c and 3c). The average lengths of chromosomes varied from 4.26 to 5.91 μm, while A_1_ and A_2_ were from 0.33 to 0.39 and 0.55 to 0.64 (Table 2).

**Table 2.** Karyomorphological characters in *Aspidistra subrotata*. ALC = average length of chromosome, NCC = number of cells calculated, A_1_ = karyotype intrachromosomal asymmetry index and A_2_ = karyotype interchromosomal asymmetry index.

Sample	Literature	Karyotype formula	ALC(μm)	NCC	A_1_	A_2_	Figure
JL	this study	2n = 76 = 44 m + 8 sm + 24 st	4.87 ± 0.03	2	0.38 ± 0.01	0.58 ± 0.01	1a, 3a
5M	this study	2n = 76 = 44 m + 8sm + 24 st	4.94 ± 0.51	3	0.35 ± 0.01	0.56 ± 0.01	1b, 3b
NG	this study	2n = 76 = 44m + 8 sm + 24 st	5.7 ± 0.30	3	0.38 ± 0.01	0.55 ± 0.01	1c, 3c
NN	this study	2n = 76 = 44 m + 8 sm + 24 st	5.56	1	0.36	0.60	
SK	this study	2n = 76 = 44 m + 8 sm + 24 st	5.06 ± 0.20	2	0.35 ± 0.00	0.58 ± 0.02	
4M1	this study	2n = 76 = 44 m + 8 sm + 24 st	4.88 ± 0.47	5	0.35 ± 0.02	0.60 ± 0.01	1d, 3d
4M2	this study	2n = 76 = 44 m + 8 sm + 24 st	4.98	1	0.37	0.64	
4M3	this study	2n = 76 = 44 m + 8 sm + 24 st	4.71 ± 0.19	5	0.36 ± 0.01	0.62 ± 0.00	
4M4	this study	2n = 76 = 44 m + 8 sm + 24 st	4.88 ± 0.09	2	0.37 ± 0.01	0.64 ± 0.02	
PM	this study	2n = 38 = 22 m + 4 sm + 12 st	5.71 ± 0.28	4	0.38 ± 0.01	0.60 ± 0.02	2a, 4a
DQ1	this study	2n = 38 = 22 m + 4 sm + 12 st	5.26 ± 0.32	4	0.37 ± 0.01	0.60 ± 0.03	2b, 4b
DQ2	this study	2n = 38 = 22 m + 4 sm + 12 st	4.47 ± 0.17	2	0.33 ± 0.01	0.56 ± 0.00	2c, 4c
BV	this study	2n = 38 = 22 m + 4 sm + 12 st	5.44 ± 0.17	2	0.37 ± 0.01	0.59 ± 0.00	2d, 4d
	Huang et al.1997	2n = 38 = 22 m + 2 sm + 14 st (2sat)	5.26	1	0.41	0.60	
~	Wang et al. 2000	2n = 38 = 22 m + 6 sm (2sat) + 10 st	5.29	1	0.35	0.59	

**Figure 2.**
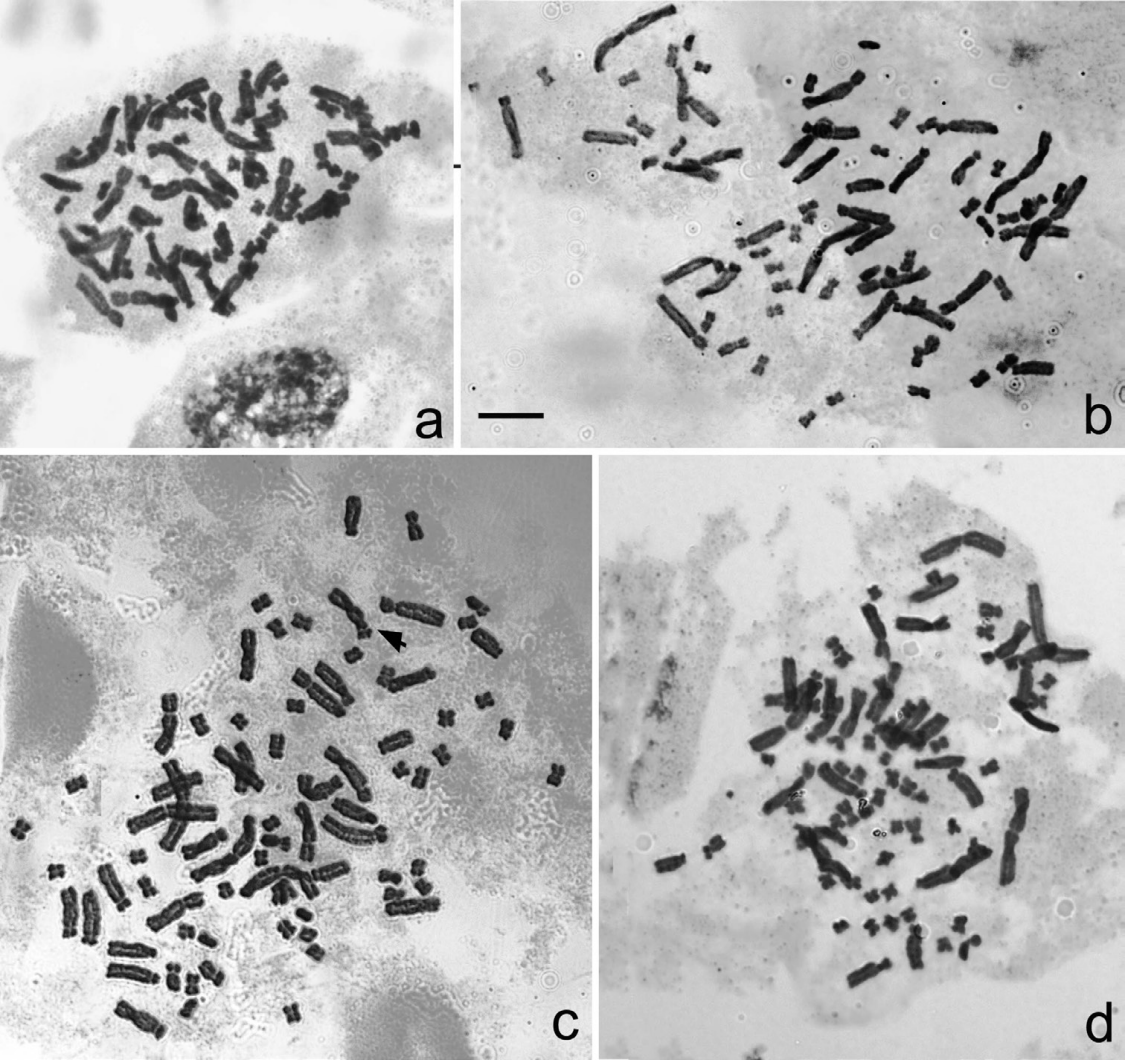
Somatic Chromosome at mitotic metaphase in *Aspidistra subrotata* of 2n = 38. **a** Mt. Poman population **b, c** Mt. Daqing population **d** Mt. Bavi population. Bar = 10 μm.

Four samples of three populations from Mt. Poman, Mt. Daqing, and Mt. Bavi of *Aspidistra subrotata* have a chromosome number of 2n = 38, uniformly formulated as 2n = 22 m + 4 sm + 12 st (Figures 2 and 4). The first and largest pair was metacentric. The pair II was larger and submedian centromeric. Chromosomes from III to VIII pair were larger [progressively smaller?] and subtelocentric. The other chromosomes were smaller, of which pair IX was submetacentric and the others were metacentric (Figure 4). No satellite was observed. The average lengths of chromosomes varied from 4.30 to 5.61 μm, while A_1_ and A_2_ were from 0.34 to 0.38 and 0.57 to 0.59 (Table 2).

**Figure 3.**
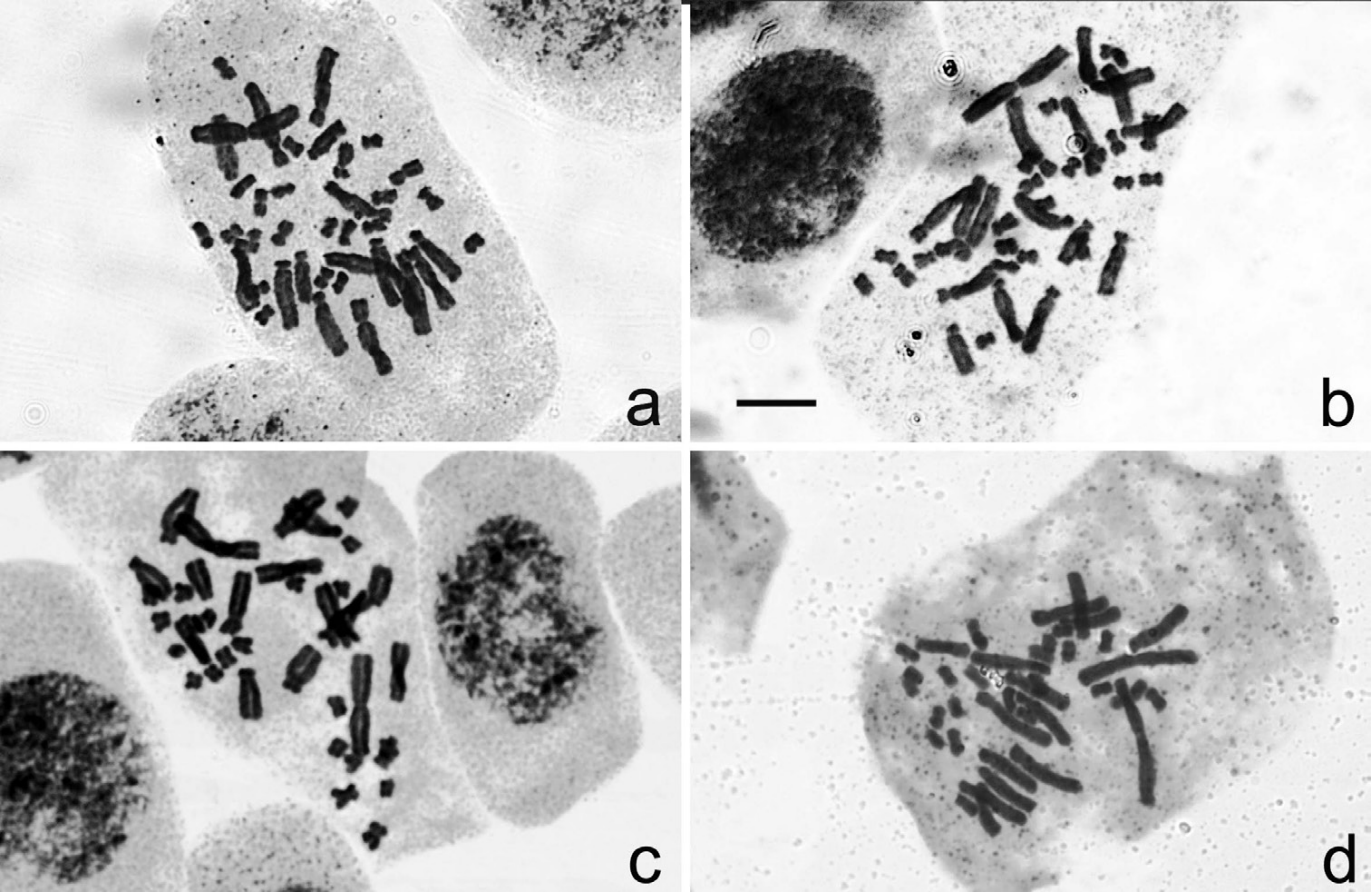
Karyotype of *Aspidistra subrotata* of 2n = 76, formulated as 2n = 44 m + 8 sm + 24 st. **a** Jilong population **b** 5th boundary marker population **c** Nonggang National Nature Reserve population, and the empty ellipse shows the presence of the secondary constriction on the chromosome 12 **d** 4th boundary marker population. Bar = 10 μm.

**Figure 4.**
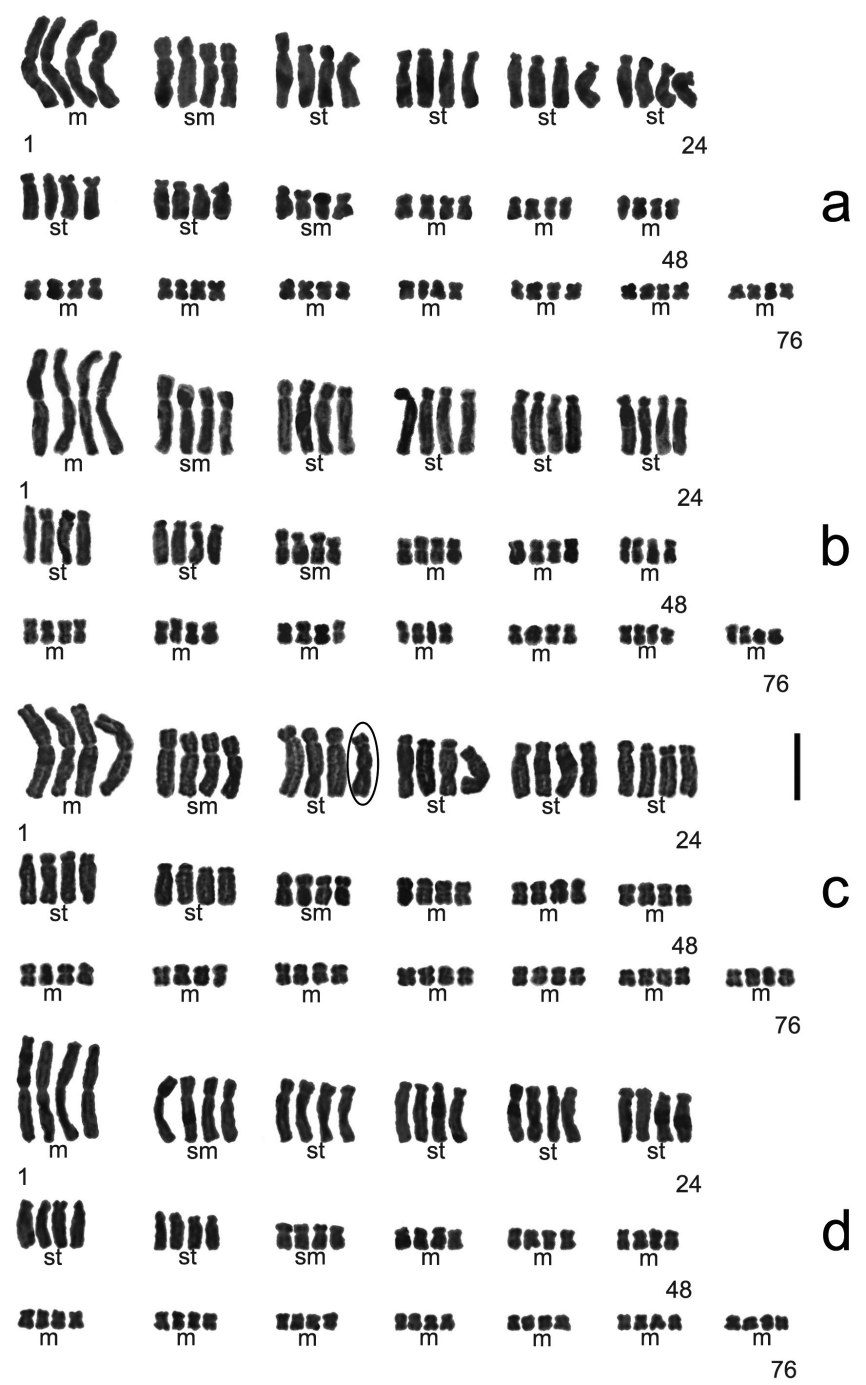
Karyotype of *Aspidistra subrotata* of 2n = 38, formulated as 2n = 22 m + 4 sm + 12 st. **a** Mt. Poman population **b, c** Mt. Daqing population **d** Mt. Bavi population. Bar = 10 μm.

### External morphology

**Figure 5.**
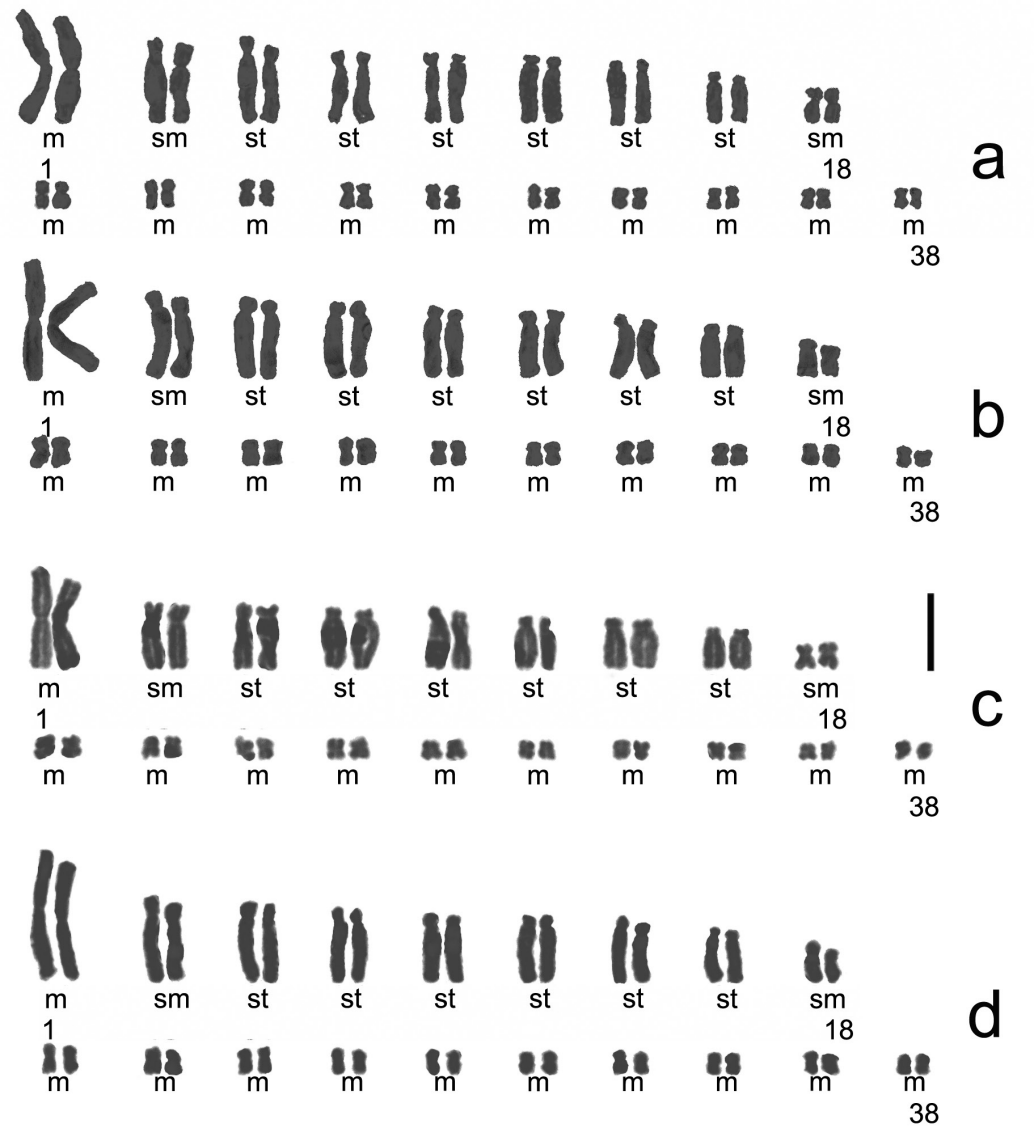
Flower morphology *Aspidistra subrotata*. **a–c** flowers of diploid plants from **a** Nonggang population **b** Nanning population **c** 4th boundary marker population **d–f** flowers of tetraploid plants from **d, e** Mt. Bavi population **f** Mt. Daqing population. Bar = 1 cm.

**Figure 6.**
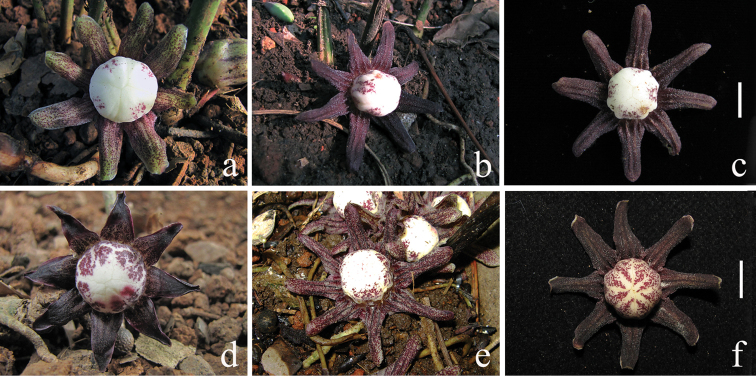
Leaf morphology of tetraploid plants of *Aspidistra subrotata*. from 4th boundary marker population. **a–c** sublinear leaves with a. smooth face **b** blotches **c** blotches and raised secondary veins **d–e** narrowly lanceolate leaves with **d** smooth face **e** blotches **f–h** ovate–lanceolate leaves with **f** smooth face **g** blotches **h** blotches and raised secondary veins **i** plants with ovate–lanceolate leaves and ones with sublinear leaves grow together.

**Figure 7.**
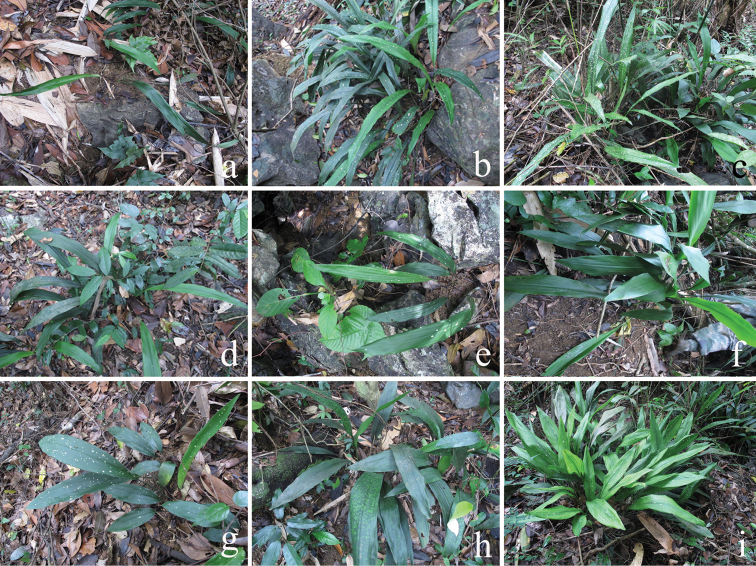
Leaf morphology of diploid plants of *Aspidistra subrotata* from Mt. Daqing population. **a–c** leaves with smooth face. a. sublinear leaves **b** lanceolate leaves **c** ovate–lanceolate leaves **d, e** ovate–lanceolate leaves with inconspicuously raised secondary veins **f** lanceolate leaves with blotches and raised secondary veins.

Based on the observation in the field, flowers of *Aspidistra subrotata* were commonly found with the perigone lobes red–purple and the stigma white with more or less small red dots on the upper surface (Figure 5). However, some variation occurs in its leaf shape, from sublinear or narrowly elliptical to ovate–lanceolate, from without blotches to with paler, often white blotches, from without raised secondary veins to with raised secondary veins in either the tetraploid population of fourth boundary marker (Figure 6) or the diploid population of Mt. Daqing (Figure 7). There were tiny differences between tetraploids and diploids; the former may be distinguished from the latter by its rigid petiole as well as thick and deep green lamina as well as its ploidy level of chromosome.

## Discussion and conclusion

### Ploidy and geographic range

The karyotypes of nine samples of six populations of *Aspidistra subrotata* are described here as tetraploid for the first time, with a chromosome number of 2n = 76 and the karyotype formulated as 2n = 44 m + 8 sm + 24 st. Among them, eight samples of five populations are all from Longzhou and are located not far away from each other (Table 1, Map 1). The secondary constriction is occasionally observed (Figure 1c and 3c). The other tetraploid sample is from the plants cultivated in Guangxi Medicine Botany Garden and is possibly the clone plants of typical *Aspidistra subrotata*. Unfortunately, the type specimen of *Aspidistra subrotata* have not been able to be checked until now. Our results may offer a hint that the plants cultivated in Guangxi Medicine Botany Garden were collected from the above county on the basis that the tetraploids so far have not been observed in any other place, and infer that the type of *Aspidistra subrotata* may be tetraploid from Longzhou. Another four samples of three populations from Mt. Poman, Mt. Daqing, and Mt. Bavi were diploid, with a chromosome number of 2n = 38 and the karyotype formulated as 2n = 22 m + 4 sm + 12 st. A difference from the previous report on the diploid states of *Aspidistra subrotata* is that no satellite was observed in the chromosomes of IX pair as sm in this study (Wang et al. 2000, Huang et al. (1997). Besides, the pair IX was reported as st in Huang et al. (1997), while one more pair of sm chromosomes occurs in Wang et al. (2000), compared with our results. The material of diploid specimens reported previously is all from cultivated plants and information about their wild population is unknown. Here, three localities are confirmed for the first time where diploid populations are distributed.

There is a long-standing debate on the ecological success of polyploids relative to diploids. Although some studies suggest that polyploids generally have larger ranges (Stebbins and Dawe 1987, Petit and Thompson 1999, Oberprieler et al. 2012), the present studies prefer to support that the correlation between ploidy and range or ecological attributes is inconsistent and appears to be taxon-specific (Martin and Husband 2009, Godsoe et al. 2013, Harbert et al. 2014). In our studies, it seems that the diploids occupy broader geographical and environmental niche spaces than the tetraploids (Map 1), which maybe offer an interesting example with which to explore the formation and the evolutionary dynamics of a new natural polyploidy complex from the limestone area of the tropical regions.

### Ploidy and external morphology

Our studies show that leaves of *Aspidistra subrotata* varied in leaf shape, color pattern, and venation in either the tetraploid population of the fourth boundary marker (Figure 6) or the diploid population of Mt. Daqing (Figure 7); the same case occurs in Phu Luang WS, north-eastern Thailand (Phonsena and De Wilde 2010), but unfortunately, the chromosome number of Thai material is unknown. As many polyploid plants are reported to be similar to their diploid parents and hence morphologically cryptic (Tate et al. 2005, Reis et al. 2014, Azizi et al. 2016), there is also tiny difference between diploids and tetraploids of *Aspidistra subrotata*. The tetraploid plants may be distinguished from the diploid plants by their rigid petioles as well as thick and deep green lamina. This type of leaf also occurs in the other three polyploid species in the genus *Aspidistra*, i.e. *Aspidistra xilinensis*, *Aspidistra cruciformis* and *Aspidistra sutepensis*, which seemly supports the notion that the polyploidy can exploit newly available habitats (Brochmann et al. 2004).

Although the leaf-shape of *Aspidistra subrotata* varies quantitatively between and within diploid or tetraploid population(s), no obvious discontinuity has been observed. It seems unreasonable to divide it into three varieties on the basis of leaf-shape. According to the independent distribution and external morphology in relation to the ploidal levels of chromosome of *Aspidistra subrotata*, two subspecies may be recognized; however, the taxonomic treatment of *Aspidistra subrotata* has not been properly dealt with until the types of all three varieties are checked and confirmed, with additional samples, geographical locations of collections, and molecular data analysis together. We hope this study will be helpful not only to better understand the origin and evolution of the species and the genus but also shed some light on the formation and the evolutionary dynamics of a new natural polyploidy complex in the limestone areas of the tropical regions.
